# Collapse from Rectal Injection

**Published:** 1889-03

**Authors:** C. Ferdinand Durand

**Affiliations:** 158 Gooding Street; Lockport, N. Y.


### *COLLAPSE FROM RECTAL INJECTION.*

By C. FERDINAND DURAND, B. A., M. B., Lockport, N. Y.

The following is a report of a case which may be of some interest and practical importance. I do not remember of having read of a similar one, but have no doubt such are sometimes met with. The facts are as follows:

W. H., a boy thirteen years old, of a nervous and excitable disposition, and who suffers from incompetency of the mitral valves, was treated by me last August for pin-worms. I saw him about 8 P. M., and gave as an enema about one pint of a solution of carbolic acid (1 in 50). After the enema he complained of dizziness, and almost immediately went out to the closet.

About ten minutes afterward he was heard moaning outside, and when carried into his room presented all the appearances of collapse.

He was unconscious; pulse, 140; respiration very feeble and extremities cold. I administered stimulants, and rubbed his arms and legs with whisky, and applied warmth. Under treatment his condition gradually improved, but it was 3 A. M. before he showed any signs of returning consciousness. He was able to be up next morning, and on the following day was sufficiently recovered to go to school, but it was several days before he returned to his usual state of health.

Such serious effects from so simple a procedure as a rectal injection were due, in my opinion, to reflex action from the irritation of Meissner's plexus.

158 Gooding Street.

